# Electrochemical sensing and quantification of theobromine in cocoa products at polyvaline functionalized graphite paste sensor electrode

**DOI:** 10.5599/admet.2928

**Published:** 2025-09-26

**Authors:** Battira Madappa Sharmila, Jamballi G. Manjunatha

**Affiliations:** Department of Chemistry, FMKMC College, Madikeri, Constituent College of Mangalore University, Karnataka, India

**Keywords:** Cyclic voltammetry, differential pulse voltammetry, electropolymerization, valine, vanillin

## Abstract

**Background and purpose:**

Theobromine (THB), an alkaloid present in various plants, is widely used in pharmaceutical formulations and food products. Increased consumption leads to health risks in both humans and animals.

**Experimental approach:**

An electrochemically polymerized polyvaline-modified graphite paste sensor (PVMGPS) was developed in this work to investigate the mechanistic and kinetic pathways of the electrooxidation of THB. Electrochemical impedance spectroscopy and scanning electron microscopy were used for characterizing the designed sensors. The fabricated PVMGPS under optimal conditions produced enhanced current responses compared to the bare graphite paste sensor (BGPS). Multiple parameters were investigated using cyclic voltammetry and differential pulse voltammetry.

**Key results:**

The electroactive surface areas of the BGPS and PVMGPS were evaluated as 0.025 and 0.252 cm^2^, respectively. The study of the effect of pH of phosphate buffer solution (PBS) and further analysis revealed that the electro-oxidation involves equivalent numbers of electrons and protons. Scan rate dependence revealed that the oxidation of THB proceeds through diffusion-controlled kinetics. The limit of detection and limit of quantification were evaluated to be 1.22 and 4.08 μM, respectively. Moreover, the voltammetric assay demonstrated a good recovery rate, proving the efficacy of the proposed sensor in detecting THB-containing food samples.

**Conclusions:**

The outcome of the analysis substantiated the efficacy, selectivity and sensitivity of the developed novel sensor for THB detection.

## Introduction

**Scheme 1.**
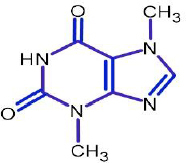
Structure of theobromine

Theobromine (THB), also known as xantheose, is a purine-based heterocyclic volatile alkaloid with a bitter taste [1]. Structurally, THB is a dimethylxanthine (3,7-dimethylpurine-2,6-dione), as shown in Scheme 1, and is copiously found in the beans of Theobroma cacao (cocoa) trees [2-4]. It is also found in small amounts in kola nuts, coffee beans, guarana and tea leaves [5,6] and used as a flavouring agent in chocolates and baked food items in the form of cocoa powder. THB finds its application in the pharmaceutical field as a neurostimulator, bronchodilator, cough suppressant, vasodilator, diuretic, anti-inflammatory, anti-tumoral, muscle relaxant, anti-depressant and mood elevator [7]. Instilled with the ability to inhibit the nucleation and growth of uric acid crystals, THB aids in the treatment of nephrolithiasis caused by uric acid and exhibits promising anticarcinogenic activity as well [8,9]. The main mechanism of the action of THB on the physiology of the human body is the inhibition of phosphodiesterases and blocking the adenosine receptors and hence can be used as an antagonist [10]. However, when consumed in higher quantities, THB induces loss of appetite, sweating, trembling, nausea, digestive issues and headaches. Consumption of chocolates causes ‘chocolate poisoning’ in dogs as the THB content causes vomiting, haematemesis, muscle twitching and polydipsia leading to lethal effects [11,12]. The detection and quantification of molecular species in food samples involves the interference of chromophore molecules present in them. Hence, a sensitive and precise detection technique becomes mandatory for the analysis [13]. There are various suggested approaches towards THB detection, such as reversed-phase high-performance liquid chromatography (RP-HPLC), microbore high-performance liquid chromatography (HPLC), ion chromatography and various other techniques [14-17]. However, these methods are expensive, laborious and time-consuming, making the analysis a complicated task to achieve [18]. Over the years, voltammetry has evolved as a powerful and versatile electroanalytical mode for rapid and effective analytical study of molecules [19]. Among numerous existing approaches, cyclic voltammetry (CV) and differential pulse voltammetry (DPV) are reliable methods for inspecting the current and voltage associated with redox reactions [20], thereby aiding in elucidating the mechanism of electron transfer in catalytic reactions [21]. Due to the rich surface characteristics, chemical inertness, broad potential window, negligibly less background current and affordability, carbon-based sensors have become a major tool in electrochemical measurements [22,23]. In addition to these attributes, these sensors provide exceptional conductivity, chemical stability, and mechanical features [24]. Carbon-based sensors, when functionalized by electropolymerization, produce a more efficient and stable tool for electroanalysis. The development of a thin layer of polymer on the sensor surface enhances electrochemical behaviour, leading to sensitive detection and effective analysis [25]. This work constitutes the utilization of an electropolymerized carbon-based sensor for THB analysis and is absolutely a novel approach with promising performance, affordability and reliability.

## Experimental section

### Chemicals and instrumentation

The reagents utilized in THB analysis were of analytical grade. THB (98 %) was procured from Tokyo Chemical Industry (Japan), valine (VL, 99 %) and vanillin (VN, 99.5 % pure) were obtained from Molychem (India). Graphite powder, silicone oil, disodium hydrogen phosphate, and monosodium dihydrogen phosphate with purities of 94, 90, 99.5 and 99 %, respectively, were purchased from Nice Chemicals, India. The required solutions for the analysis were formulated using double-distilled water (DW) obtained from the VITSIL-VBSD/VBDD water purifying unit. CV and DPV measurements were performed using a CHI-6038E (USA) electrochemical analyser equipped with three electrodes. A bare graphite paste sensor (BGPS) or a polyvaline-modified graphite paste sensor (PVMGPS) served as the working electrode, a calomel electrode as the reference electrode, and a platinum wire as the counter electrode. The characterization of the sensor material was performed with a scanning electron microscopy (SEM) instrument available at Vijnana Bhavan, Mysore University.

### Preparation of real sample solution

Cocoa powder used in culinary preparations and dark chocolate were bought from the local grocery store. The measured amounts of these samples were dissolved in the required amount of dimethyl sulfoxide, followed by filtration with Whatman filter paper. The necessary solutions for the analysis were prepared by diluting the obtained filtrate using DW.

### Fabrication of sensor

The graphite powder and silicone oil in a 70:30 ratio was blended well to achieve a well-mixed paste. The resultant paste was compactly filled into the cavity of the Teflon tube (3 mm in diameter). BGPS with an even surface was attained by smoothening on a soft tissue. To attain an electrical connection, a copper wire was passed through the Teflon tube. Further, the BGPS was subjected to electropolymerization to develop the desired PVMGPS.

### Electropolymerization and cycle optimization

**Figure 1.**
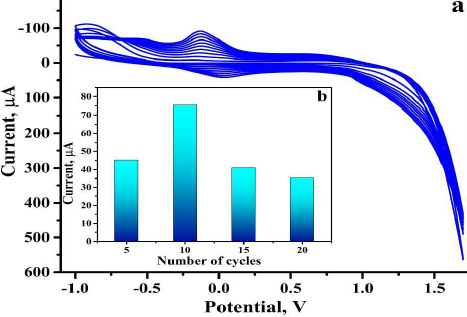
(a) CV response of 1 mM VL in 0.2 M PBS at a scan rate of 0.1 V s^-1^, (b) plot depicting current variation with number of sweep cycles

The BGPS was modified via electropolymerization with VL as monomer by employing CV technique at a scan rate of 0.1 V s^-1^ in the potential window of -1.5 to 2.0 V. CV was recorded for 1 mM valine in 0.2 M phosphate buffer solution (PBS) of pH 5.5 as supporting electrolyte at the surface of BGPS at 5, 10, 15 and 20 sweep cycles (as illustrated in Figure 1a). The number of sweep cycles significantly affects the accumulation of VL monomers on the sensor surface. The appropriate deposition of the monomers forms a thin layer of polyvaline on the sensor surface, which enhances the electrochemical performance of the developed sensor. The sweep cycle at which the BGPS generated a comparatively high oxidation peak current was considered the optimum cycle for the analysis. As illustrated in Figure 1b, the sweep cycle of 10 has generated a higher peak current value than 5, 15 and 20 sweep cycles and hence is considered as an optimal sweep cycle for further analysis.

## Results and discussion

### Interpretation of the morphology of the sensor material

**Figure 2.**
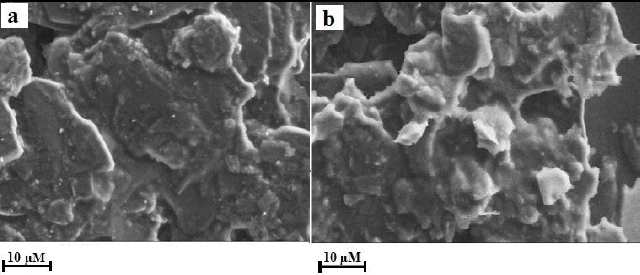
SEM images (a) BGPS and (b) PVMGPS

The morphology of the sensors was assessed using SEM, a technique that enables the acquisition of images of the sensor material by scanning its surface with a high-energy electron beam [26]. As depicted in Figure 2, the structural data obtained by SEM exemplifies a seeming variation in the morphology of BGPS and PVMGPS. The PVMGPS shows the formation of a thin layer of VL monomers, which are depicted as white, spongy-like structures on the surface. This confirms that electropolymerization has induced these morphological changes, resulting in the elevated voltammetric response of PVMGPS in the analysis of THB.

### Evaluation of electroactive surface area and EIS study

**Figure 3.**
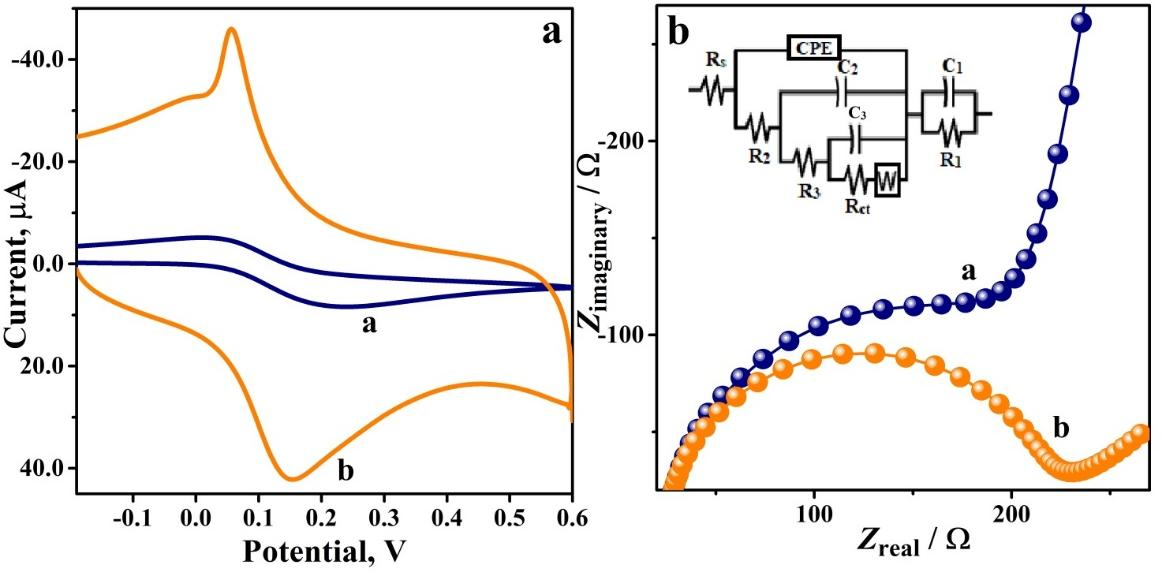
(a) CV depicting the peak current responses of 1.0 mM K_4_[Fe(CN)_6_] in 0.1 M KCl at BGPS (curve a) and PVMGPS (curve b), (b) Nyquist plot of BGPS (semi-circle a) and PVMGPS (semi-circle b) with the equivalent electrical circuit

The electroactive surface area describes the exterior region available for the electrochemical activity of electroactive species on the surface of the sensor and hence explains its sensing efficiency. CV was recorded for K_4_[Fe(CN)_6_] (1.0 mM) in 0.1 M KCl supporting electrolyte at a scan rate of 0.1 V s^-1^ on BGPS and PVMGPS surfaces. The results of the analysis are depicted in Figure 3a, which displays the variance in the redox current responses and apparent redox potential peak separation. The obtained anodic and cathodic peak current values are 8.42 and 5.10 μA for BGPS, and 42.40 and 45.97 μA for PVMGPS, respectively. The corresponding peak separation (Δ*E*_p_) values for BGPS and PVMGPS are 0.22 and 0.09 V, respectively. The comparatively higher redox peak current values and the decreased Δ*E*_p_ for PVMGPS evidently verify that the electrochemical modification has improved the sensing capacity of the electropolymerized sensor.

Electrochemical impedance spectroscopy (EIS) offers a precise and effective method for evaluating the conductivity and resistivity of electrochemical systems [27].

**Figure 4.**
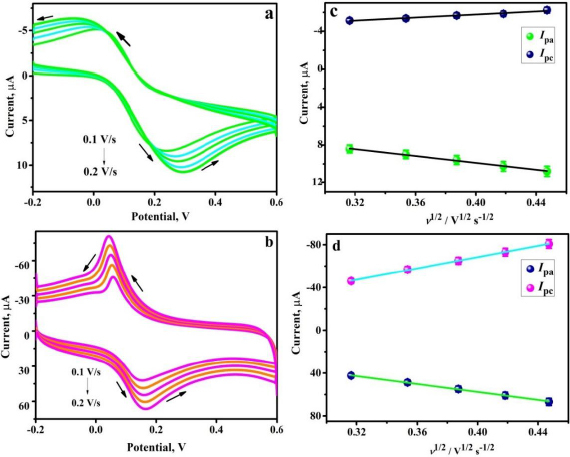
CV response of 1mM K_4_[Fe(CN)_6_] in 0.1 M KCl at varied scan rate: (a) BGPS electrode, (b) PVMGPS electrode; (**c**) plot of redox peak current *vs. ν* ^1/2^ for BGPS, and (d) plot of redox peak current *vs. ν* ^1/2^ for PVMGPS

EIS analysis was performed for 1.0 mM K_4_[Fe(CN)_6_] in a suitable electrolyte (0.1 M KCl) at a DC potential of 0.05 V and an amplitude of 5 mV in the frequency range of 1.0 Hz to 1000 kHz. The Nyquist plot in Figure 3b, comprising both semicircular and linear components, appears to display the variation in the radii of the semicircles and the charge transfer resistance (*R*_ct_) values for BGPS and PVMGPS. The data obtained in the EIS analysis was fitted into an equivalent electric circuit (shown in the inset of Figure 3b) comprising components such as *R*_s_ (solution resistance), CPE (constant phase element), Warburg impedance (*W*) and *R*_ct_. The *R*_ct_ values obtained were 299 and 234 Ω for BGPS and PVMGPS, respectively. The smaller semicircle and the lower *R*_ct_ value observed for PVMGPS contribute to its superior electrocatalytic performance by enabling faster electron transfer. Further, the surface areas of BGPS and PVMGPS were estimated by recording CV for 1 mM K_4_[Fe(CN)_6_] in the supporting electrolyte (0.1 M KCl) at varied scan rates (0.1 to 0.2 V s^-1^). The anodic and cathodic peak currents (*I*_pa_ and *I*_pc_, respectively) obtained for different scan rates for BGPS and PVMGPS are depicted in Figures 4a and 4b, respectively.

It is noticed that the potential of the oxidation and reduction peaks shifts toward positive values and negative values, respectively, in both sensors. Figures 4c and 4d demonstrate the graph of redox peak current *vs. ν* ^1/2^, signifying the linear relationship of the peak current with the scan rate at BGPS and PVMGPS surfaces.

The linear regression equation (LRE) for *I*_pa_ *vs. ν* ^1/2^ can be stated by Equations (1) and (2):



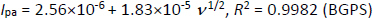

(1)




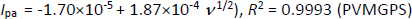

(2)


Substituting the slope from Equations (1) and (2) in the Randles-Ševčik Equation (3), the active surface area was evaluated.



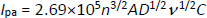

(3)


Here, *I*_pa_ is the anodic peak current, *n* = 1 is the number of electrons, and *D* is the diffusion coefficient (7.6×10^-6^ cm^2^ s^-1^), *A* is the surface area, *ν* / V s^-1^ is the scan rate and *C* / mol cm^-3^ is the concentration of K_4_[Fe(CN)_6_]. The surface area was computed as 0.025 cm^2^ for BGPS and 0.252 cm^2^ for PVMGPS, which signifies that the modification of BGPS via electropolymerization has enhanced the active surface area and hence the active sites necessary for efficient electrochemical activity.

### Influence of the pH of supporting electrolyte

**Figure 5.**
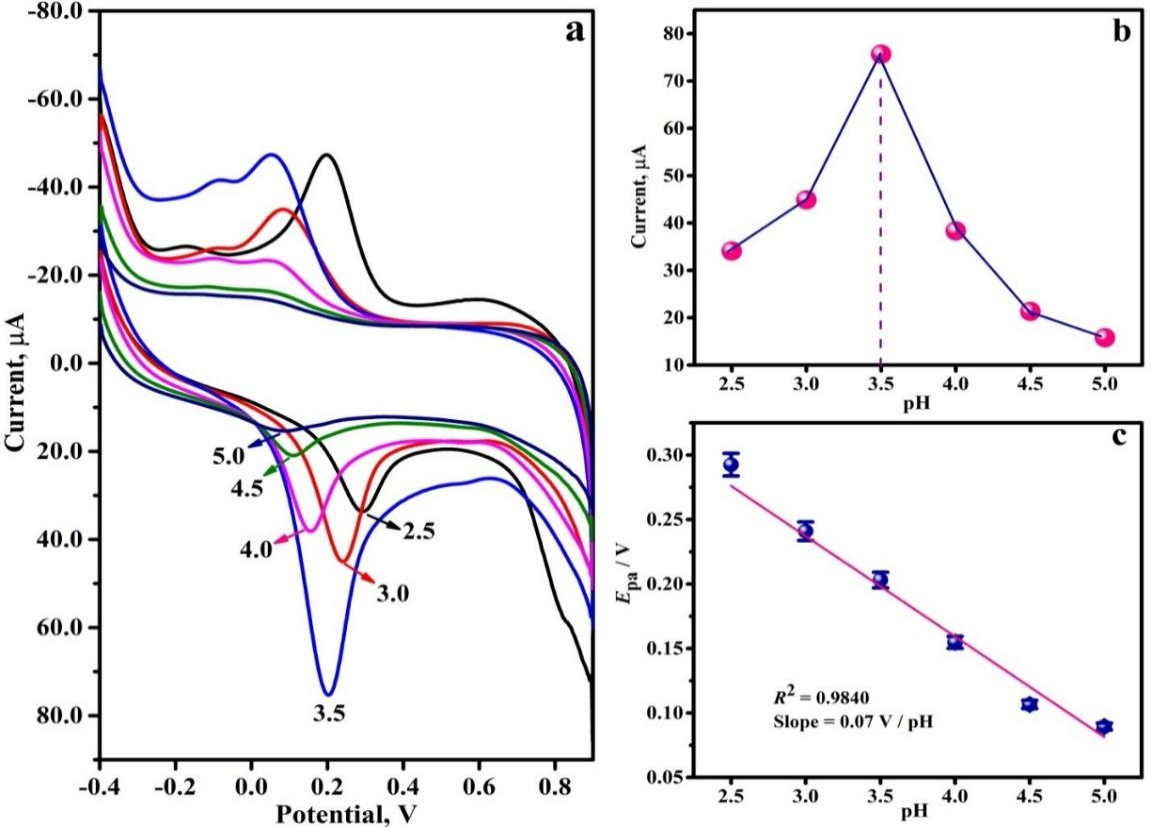
(a) CVs of 0.01 mM THB at varied pH from 2.5 to 5.0, (b) graph displaying the variation of anodic peak current with altered pH range, (c) oxidation peak potential (*E*_pa_) *vs.* pH plot

The influence of the pH of the supporting electrolyte (0.2 M PBS) on the electrochemical activity of 0.01 mM THB on the PVMGPS surface was studied with CV. CVs were recorded for THB in 0.2 M PBS at pHs ranging from 2.5 to 5.0. The redox peaks obtained at a varied pH range are presented in Figure 5a. It is observed that at a pH of 3.5, a well-defined anodic peak with a current value of 75.66 μA is obtained, and the oxidation peaks diminish at other pH values (as evident in Figure 5b). This substantiates the maximum redox activity of THB at the PVMGPS surface at a pH of 3.5; hence, this pH was considered the optimum for further analysis.

A graph of different pH values was plotted against oxidation peak potential (*E*_pa_), illustrating the negative linear shift of *E*_pa_ with increasing pH from 2.5 to 5.0, as depicted in Figure 5c. The linear regression equation can be represented by Equation (4):



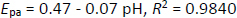

(4)


The slope value of 0.07 V/pH from Equation (4) validates that the THB undergoes electro-oxidation through the transfer of the same number of protons and electrons [28,29].

### Electrochemical response of theobromine at the surfaces of the bare graphite paste and polyvaline-modified graphite paste sensors

The electrochemical interaction between the developed sensor and the target analyte plays a prominent role in the redox reaction and, consequently, in the redox mechanism. The sensing capacity of BGPS and PVMGPS was compared by recording the CV for 0.01 mM THB in PBS (0.2 M, pH 3.5) at BGPS and PVMGPS surfaces at a scan rate of 0.1 V s^-1^.

**Figure 6.**
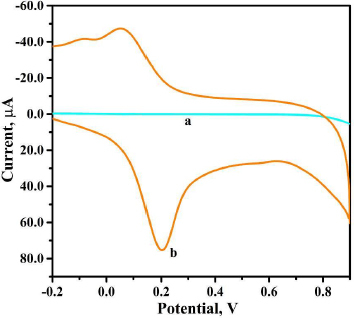
CV of 0.01 mM THB in 0.2 M PBS at pH 3.5 at BGPS (curve a) and at PVMGPS (curve b)

Figure 6 clearly indicates that the PVMGPS exhibits enhanced electrochemical performance, with an anodic peak current value of 75.66 μA (curve b), compared to BGPS. The evident disparity in the obtained anodic current responses clearly emphasizes the advantage of modifying the carbon electrode with the electropolymerized polyvaline in achieving a sensor with improved voltammetric performance, producing a high peak current value.

### Influence of accumulation time

**Figure 7.**
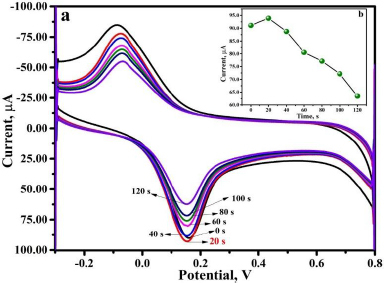
(a) CV representing the 0.01 mM THB response at different accumulation time intervals, (b) plot of current *vs.* time

The current sensitivity and peak current values are significantly influenced by the accumulation of the target analyte on the sensor surface. To optimize the time at which maximum THB accumulation takes place at the surface of PVMGPS, CV recordings were taken for 0.01 mM THB at optimum experimental conditions at various accumulation time intervals (0 to 120 s) as depicted in Figure 7a.

The oxidation peak current vs. time interval was plotted (Figure 7b), and it is apparent that at 20 s, the oxidation current response is maximum. The intense electrochemical response can be attributed to the adequate accumulation of THB at the PVMGPS surface compared to the other considered time intervals. Hence, for every CV recording in the analysis, an accumulation time of 20 s was maintained to achieve the best results.

### Impact of scan rate

**Figure 8.**
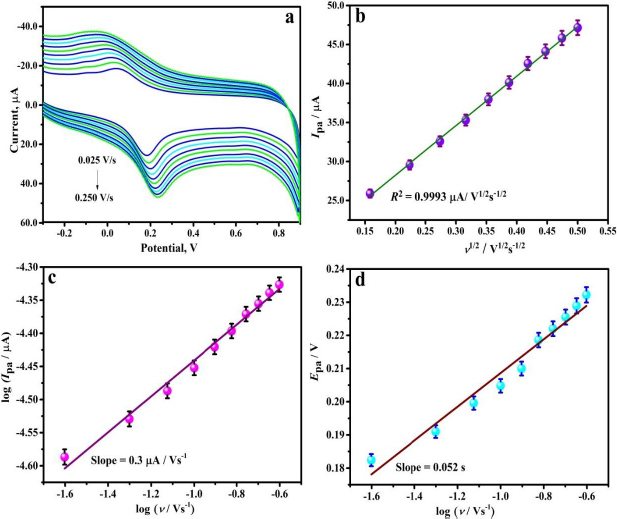
(**a**) CV response of 0.01 mM THB in PBS (0.2 M PBS) at varied scan rate, (**b**) plot of *I*_pa_ *vs. ν* ^1/2^, (**c**) graph of log *I*_pa_ *vs.* log *ν*, (**d**) plot of *E*_pa_ *vs.* log *ν*



The CV method was employed to investigate the electrochemical behaviour of 0.01 mM THB on the PVMGPS surface at varied scan rates. Furthermore, with the obtained data, the mechanism of the oxidation process was analysed. CV measurements were done for THB in 0.2 M PBS (of optimum pH of 3.5) at varied scan rates (0.025 to 0.250 V s^-1^) in the potential range of -0.4 to 0.9 V. From Figure 8a, it is noticeable that the redox peak current increases as the scan rate is increased from 0.025 to 0.250 V s^-1^. Figure 8b depicts the existing linearity between the oxidation peak current (*I*_pa_) and the square root of scan rate (*ν*^1/2^) with a regression coefficient (*R*^2^) of 0.9993. The relevant linear regression equation (LRE) can be stated as in Equation (5):

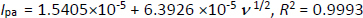

(5)


Further, the linear relationship obtained by plotting the graph of log of anodic peak current and log of scan rate (as illustrated in Figure 8c) can be expressed by Equation (6):



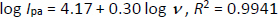

(6)


The linearity between *I*_pa_ and *ν*^1/2^ with *R*^2^ = 0.9993 observed in Figure 8b and the slope value of 0.3 from Equation (6) (corresponding to Figure 8c) validates the diffusion-controlled kinetics involved in the electrooxidation of THB at PVMGPS surface [30,31]. The number of electrons participating in the oxidation of THB on the PVMGPS surface was evaluated by substituting the slope obtained from the plot of *E*_pa_ *vs.* log *ν* (Figure 8d) in Laviron’s equation. The LRE for the linear dependence of *E*_pa_ on log *ν* can be stated as in Equation (7):



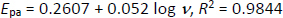

(7)


The slope value of 0.052 from Equation (7) is substituted into Laviron’s Equation (8):



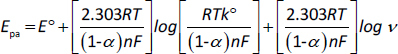

(8)


Here, *R*, *T* and *F* signify universal gas constant, absolute room temperature and Faraday’s constant, respectively. *α* and *k*^o^ denote the charge transfer coefficient and heterogeneous rate constant, respectively. The number of electrons (*n*) computed was equal to two, which confirms the involvement of two electrons in the electrooxidation of THB at the PVMGPS surface.

**Scheme 2.**
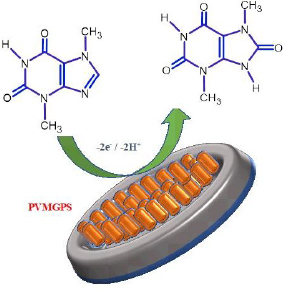
Oxidation mechanism of THB at PVMGPS surface

The feasible mechanism of the reaction has been illustrated in Scheme 2.

### Variation of theobromine concentration and its impact on peak current

**Figure 9.**
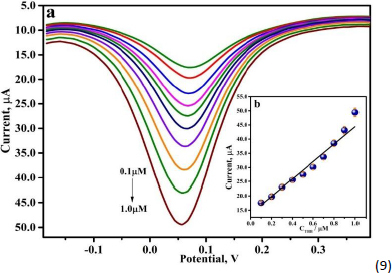
(a) DPV of various concentrations of 0.01 mM THB in 0.2 M PBS, (b) calibration plot of 0.01 mM THB in 0.2 M PBS at different concentrations



DPV technique, at optimum experimental conditions, was employed to analyze THB in various concentrations at the fabricated PVMGPS surface in 0.2 M PBS. As demonstrated in Figure 9a, as the concentration of THB was augmented from 0.1 to 1.0 μM, the anodic peak current values increased. Acceptable linearity with a correlation coefficient of 0.9869 was observed in the calibration plot (Figure 9b), and the linear relationship between *I*_pa_ and concentration of THB (C_THB_) can be expressed in LRE, Equation (9):

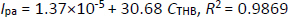

(9)


Evaluation of limit of detection (LOD) and limit of quantification (LOQ) was performed by substituting the slope from Equation (9) in LOD = 3 *S*/*N* and LOQ = 10 *S*/*N*, respectively, where *S* is the standard deviation of the blank solution (0.2 M PBS of pH 3.5) and *N* is the slope of the calibration plot. The corresponding values obtained for LOD and LOQ were 1.22 and 4.08 μM. Furthermore, the electroanalytical performance of the developed sensor was compared with the THB analyses published and is illustrated in Table 1.

**Table 1.** Comparison of LOD values with related electroanalytical methods

Detection technique	Sensor	LOD, μM	Linear range, μM	Ref.
DPV	BDDE^4^	0.42	0.99-54.5	[32]
SWV^1^	BDDE	0.51	0.99-54.5	
DPV	βH-MnO_2_-NF/GCE^5^	0.008	0.01-320	[33]
SWV	BDDE	0.025	0-50	[34]
AdSV^2^	GCE^6^	22	1-30	[35]
CV	CPE@[V II O(salen)]^7^	0.022	-	[36]
SWV	CPE@[V II O(salen)]	0.4	-	
LSV^3^	CMC-fMWCNTs/GCE^8^	0.21	0.5-80	[37]
Amperometry	Screen-printed carbon electrode	11.4	20-400	[38]
DPV	PVMGPS	1.22	0.1-1.0	This work

^1^SWV - square wave voltammetry

^2^AdSV- adsorptive stripping voltammetry

^3^LSV - linear sweep voltammetry

^4^BDDE - boron-doped diamond electrode

^5^βH-MnO_2_-NF/GCE - β-type hierarchical structure of the MnO_2_ nano-flowers modified glassy carbon electrode

^6^GCE - glassy carbon electrode

^7^CPE@[V II O(salen)]- carbon paste electrode modified with vanadium (II) oxide salen complex

^8^CMC-fMWCNTs/GCE – carboxymethylcellulose functionalized multiwalled carbon nanotubes modified glassy carbon electrode

When compared to the present work, the other reported works employed relatively expensive electrode materials in the voltammetric analysis of THB. The obtained LOD value of 1.22 μM in the present analysis verifies the efficiency of PVMGPS, which is accompanied by affordability and eco-compatibility, in comparison with other sensors for the detection of THB.

### Real sample analysis

**Figure 10.**
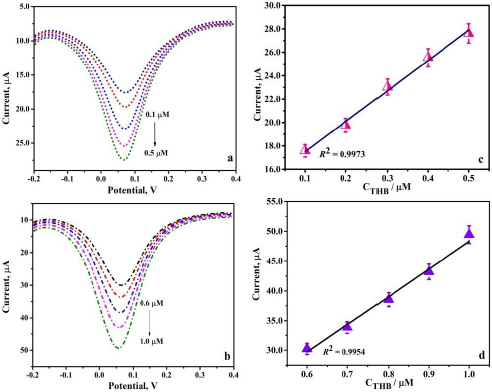
(a) DPV response of cocoa powder solution (0.1-0.5 μM in 0.2 M PBS), (b) DPV response of dark chocolate sample (0.6-1.0 μM in 0.2 M PBS), (c) and (d) corresponding calibration plots

To substantiate the real-world application of the fabricated PVMGPS, its sensing capacity was assessed using food samples containing THB. The necessary solutions of the food samples were prepared, and DPV was recorded at optimal experimental conditions using the standard addition method. As depicted in Figures 10a and 10b, the cocoa powder and dark chocolate samples provided convincing results demonstrating relevant anodic peak currents at varied concentrations of 0.1-0.5 and 0.6-1.0 μM, respectively.

To authenticate the obtained data, using the calibration graphs (Figures 10c and 10d), LOD was calculated for both the food samples and the results obtained were 1.4 and 0.8 μM, respectively.

Additionally, the rate of recovery was evaluated, and the relevant outcome of the analysis is represented in Table 2, portraying an excellent recovery rate in both samples. Thus, based on the analysis, it can be concluded that the fabricated PVMGPS can be used as an effective sensor for THB analysis in various food samples, exhibiting appreciable sensitivity and recovery rates.

**Table 2.** Representation of recovery rates for the food samples

Food sample	Content, μM		Recovery, %
	Added	Found	
Cocoa powder	0.1	0.099	99.83
	0.2	0.199	99.94
	0.3	0.299	99.91
	0.4	0.399	99.88
	0.5	0.496	99.71
Dark chocolate	0.6	0.599	99.83
	0.7	0.698	99.67
	0.8	0.799	99.97
	0.9	0.898	99.84
	1.0	0.999	99.98

### Simultaneous analysis, interference study and assessment of miscellaneous attributes of polyvaline-modified graphite paste sensor

**Figure 11.**
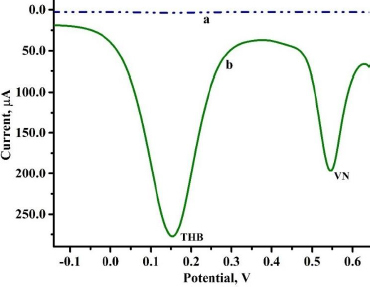
DPV response representing the concurrent analysis of THB and VN at BGPS (curve a) and PVMGPS (curve b)

The concurrent analysis of the target analyte with species with similar electrochemical activity helps to comprehend the efficiency of the fabricated sensor. DPV recordings were made for 0.01 mM THB with VN of the same concentration in PBS (0.2 M, pH 3.5) at 0.1 V s^-1^ scan rate. The oxidation peak current values obtained for BGPS (curve a) and PVMGPS (curve b) were compared. It is observed in Figure 11 that BGPS barely divulges a significant electrochemical response in contrast to the developed sensor. PVMGPS exhibits an enhanced anodic current response for THB and VN, thereby substantiating its electrochemical sensing capability in the presence of two electroactive species.

To assess the electrochemical performance of the proposed sensor in the presence of the interfering species, the CV technique was employed. CV measurements were made for 0.01 mM THB at optimal conditions in the presence of various interfering metal ions such as Cu^2+^, K^+^, Pb^2+^, Cd^2+^, Na^+^, Mg^2+^ and vitamins such as B_6_ and B_12_.

**Figure 12.**
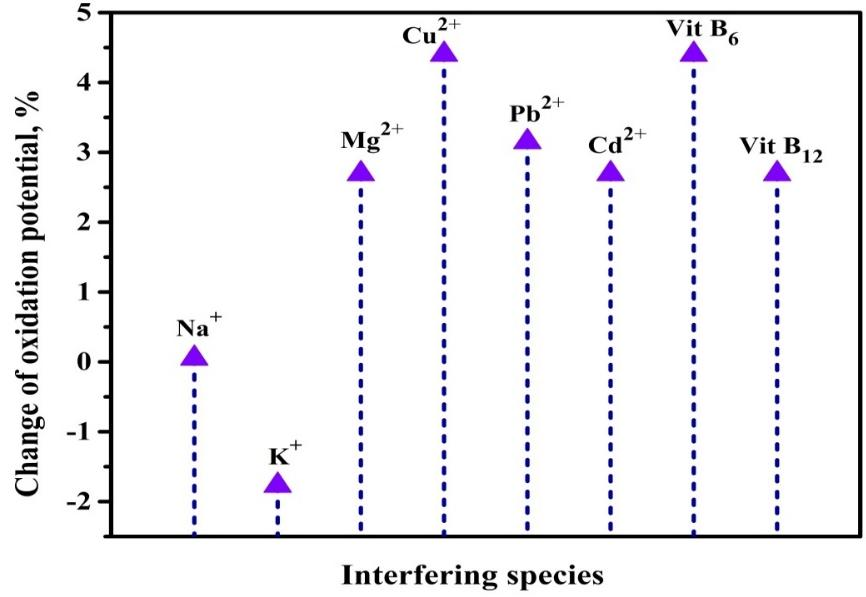
Representation of the change of oxidation potential in the presence of metal ions and vitamins

The addition of these interferents had a negligible impact on the electrochemical activity of the PVMGPS, with a potential variation of less than ±5%, as depicted in Figure 12. This demonstrates that the THB sensing ability of PVMGPS remains unchanged even in the presence of interfering species, thereby authenticating the selectivity of the developed sensor towards the target analyte.

The repeatability, reproducibility, and stability of the PVMGPS were evaluated by recording CV for 0.01 mM THB in PBS (0.2 M, pH 3.5) at a scan rate of 0.1 V s^-1^. CV measurements recorded for the same fabricated sensor five times (repeatability) presented RSD of 4.88 % (*n*=5) and for the five newly prepared sensors (reproducibility) demonstrated RSD of 4.92 % (*n*=5). The stability of PVMGPS was evaluated by recording the oxidation peak current value on the first and last day of analysis. The comparison of the peak current values showed a current retention of 98 % of the initial current, demonstrating a high degree of stability. The outcome of these analyses clearly verifies that the developed PVMGPS is an appropriate sensor for THB analysis, offering high stability and performance.

## Conclusion

In this present work, an electrochemical sensor was developed for the analysis of THB with remarkable sensitivity and efficacy. To achieve improved electroanalytical features, the electropolymerization technique was employed, using VL as a modifier for BGPS. The structural features of the working sensors were evaluated with SEM and EIS techniques. The electrochemical response of THB at BGPS and PVMGPS surfaces was compared, and the modified sensor proved its efficacy with a significant enhancement in the oxidation peak current value. The pH assay of THB in 0.2 M PBS revealed the optimum as 3.5 for the analytical parameters. It was observed that the mechanism of THB oxidation proceeded via diffusion-controlled kinetics, involving the transfer of two electrons. The fabricated sensor demonstrated an acceptable LOD and LOQ value of 1.22 and 4.08 μM, respectively. The stability, reproducibility and repeatability of the designed sensor provided a convincing outcome, substantiating the reliability for THB analysis. The analysis of food samples containing THB validated the practical application of the sensor, yielding high recovery rates, and thus proved PVMGPS to be a reliable and affordable sensor.
